# Towards universal social protection for people affected by tuberculosis in the Western Pacific Region: a social protection baseline assessment and policy entry points

**DOI:** 10.1186/s41182-025-00887-2

**Published:** 2026-03-12

**Authors:** D. Boccia, K. Rahevar, D. J. Carter, J. M. Pescarini, A. Schwalb, T. Islam, K. H. Oh, F. Morishita, R. P. Yadav

**Affiliations:** 1 Faculty of Epidemiology and Population Health, London School of Hygiene and Tropical Medicine, London, UK; 2 World Health Organization Regional Office for the Western Pacific, Manila, Philippines; 3 Faculty of Population Health and Policy, London School of Hygiene and Tropical Medicine, London, UK; 4 Centro de Integracao de Dados e Conhecimentos Para Saude, Instituto Goncalo Moniz, Fundacao Oswaldo Cruz, Salvador, Brazil; 5 TB Modelling Group, TB Centre, London School of Hygiene and Tropical Medicine, London, UK; 6 Instituto de Medicina Tropical Alexander von Humboldt, Universidad Peruana Cayetano Heredia, Lima, Peru; 7 Department for HIV, TB, Hepatitis and STIs, World Health Organization, Geneva, Switzerland

## Abstract

**Background:**

Achieving universal social protection (SP) coverage for people affected by tuberculosis (TB) is increasingly recognised as an essential component of its response, as well as other diseases of poverty. Realising this goal requires to clearly understand the SP needs of people affected by TB and to identify means to maximise their access to existing or new SP benefits in an efficient, effective, and sustainable manner.

**Main body:**

To address these questions, between 2022 and 2023, the WHO Western Pacific Regional office conducted the first SP baseline assessment for people affected by TB in Mongolia, Lao People’s Democratic Republic, the Philippines, Cambodia, and Viet Nam. This exercise encompassed a desk review of SP programmes operating in these countries, followed by an expert consultation to discuss barriers and entry points to expand SP coverage among people affected by TB. Overall evidence gathered from publicly available reports and publications suggests that existing SP programmes in these countries are insufficiently accessible and inadequate to meet the needs of people affected by TB. Most countries provide TB-specific benefits only to people with multidrug-resistant TB, leaving most people with TB unserved. The most reported barriers to access to SP included lack of awareness, stigma, poverty, as well as programmes’ fragmentation, and administrative and financial constraints. Identified solutions included raising awareness about SP, extending TB-specific SP benefits to all people with TB in need, advocating for a better inclusion of people with TB into existing governmental programmes, and strengthening the referral system across the health and SP sectors.

**Conclusions:**

By identifying concrete policy entry points and actionable solutions, this SP baseline assessment provided a foundation for these five countries to embed social protection more systematically into their national TB responses. Ideally, this effort should now be replicated in all high TB-burden countries willing to achieve universal SP coverage among people affected by TB. The lessons that emerged from this baseline assessment are consistent with the recommended actions and principles underlying the Western Pacific Regional Framework for Reaching the Unreached and are thus transferrable to other diseases of poverty.

**Supplementary Information:**

The online version contains supplementary material available at 10.1186/s41182-025-00887-2.

## Background

An increasingly solid base of evidence supports the role of social protection (SP) in the prevention, care, and control of diseases of poverty such as tuberculosis (TB) [1, 2]. This evidence also forms the basis of the new target approved during the High-Level Meeting of the United Nations General Assembly on the fight against TB, according to which by 2027 all people with TB will have to have access to a comprehensive package of health and social benefits [3]. The importance of universal SP coverage among people affected by TB to strengthen the response to the epidemic at the regional level is also fully acknowledged in the Western Pacific Regional framework to End TB (2021–2030) [4]. In 2017, key stakeholders from the six high TB-burden countries in the region discussed actions towards Universal Health Coverage (UHC) and SP in the Western Pacific Region (WPR), recommending the establishment and operationalisation of a research platform to better understand the main barriers and opportunities to progress towards UHC and maximise the use and impact of SP programmes [5].

In 2022–2023, the WHO Western Pacific Region responded to this call by conducting a SP baseline assessment in five high TB-burden countries in the region, including Mongolia, Lao People’s Democratic Republic (PDR), the Philippines, Cambodia, and Viet Nam. This paper discusses the key findings and lessons that emerged from this exercise and how they can inform future actions to expand SP coverage among people affected by TB and ultimately strengthen the TB response in these countries.

## Methodological approach

This social protection baseline assessment aimed to evaluate the extent to which existing TB-sensitive and TB-specific programmes (Box 1) operating in Mongolia, Lao People’s Democratic Republic (PDR), the Philippines, Cambodia, and Viet Nam were inclusive for people affected by TB and responsive to their needs. The approach taken in these five countries largely draws from the content of the recently published WHO-ILO Guidance on Social Protection for People Affected by TB [6]: specifically, between 2022 and 2023, in each country a structured desk review was conducted, followed by a multisectoral expert consultation. The desk reviews examined national laws, programme documents, and grey literature to identify SP programmes entitlements, coverage, eligibility rules, and implementation features. Findings were triangulated with insights from country consultations involving representatives from health, social protection, and civil society sectors to discuss the supply- and demand-side barriers (Table 1) limiting access for people affected by TB to the identified SP programmes and ultimately formulate solutions to make them more accessible and impactful. Further methodological details are available in Additional file 1: Annex 1.

**Box 1:** Definition of TB-sensitive and TB-specific social protection programmes

*TB-sensitive* programmes are programmes not targeted specifically at households or individuals affected by TB. While not designed intentionally to address any TB-related objective, these programmes can potentially affect the TB epidemic by including among their eligibility criteria people with, at risk of, and/or affected by the consequences of TB. Typically, these programmes are not initiated by TB programmes and are not under the direct control and management of the health sector.	
*TB-specific* social protection programmes are programmes exclusively addressed to people with TB and their households. Typically, these programmes are initiated by and are under the responsibility of TB programmes and are intended to respond to specific needs not covered by the mainstream social protection system. Such programmes have the explicit objective of reducing TB incidence, mortality and patient costs by mitigating the impact of poverty, vulnerability and social exclusion on households and individuals affected by TB.

**Table 1:** Examples of demand-side and supply-side barriers to access to social protection experienced by people affected by TB

Type of barrier	Category	
	Demand-side	Supply-side
Administrative/Implementation	People struggle to get enrolled because of administrative complexities and constraints as lack of documentation, lack of bank account, citizenship status, etc.	Benefits are delivered with delays or are insufficient to meet the needs of people affected by TB Eligible people are mistakenly excluded by the programme The programme does not cover areas where TB is most endemic in the country
Distance-related	People live too far from the point of enrolment or delivery of benefits	The enrolment and delivery of benefits is centralised and distant from the areas were people mostly in need live
Lack of knowledge/awareness	People are unaware of the existence of social protection programmes available to them, ignore their eligibility and/or the administrative requirements to access the benefits they are entitled to	There is no clear or transparent information and communication on how to access the available social protection benefits
Structural and social determinants	People cannot afford hidden or up-front costs or opportunity costs (i.e. enrolment fees, transport costs, income loss) Illiteracy, language barriers Living in geographically isolated areas	Limited fiscal space and/or political will to make existing programmes more TB-sensitive (i.e. limited room or will to expand coverage, eligibility criteria and/or increase the size of the benefits)
Stigma	People fear to be stigmatised either because of their TB or beneficiary status	People affected by TB are stigmatised by social workers in charge of the enrolment or delivery because considered contagious and/or underserving of support
Health related	People are too sick (or suffer from disabilities) to seek information and/or start the enrolment process into existing programmes	Social protection services providers are unaware of the symptoms and clinical needs of people affected by TB

Ethical approval was not required as only secondary and anonymised information was used.

## The emerging social protection landscape

Countries involved in this SP baseline assessment varied in terms of programme availability, current SP effective coverage, and SP expenditures [7]. However, all of them offered some form of TB-sensitive and TB-specific programmes (Additional file 2: Appendix 2). The key features of the identified TB-sensitive programmes are summarised in Additional file 3: Appendix 3. Overall, the appraisal of these programmes and associated literature revealed that data on coverage levels among people with TB were limited and, when available, suggested that just a minority of people with TB (even when technically eligible) had access to social protection benefits. For instance, in Lao PDR, only 0.4% of people with TB reported having access to any type of social welfare [8]. Likewise, in the Philippines, only 1.3% of TB-affected households were receiving the national cash transfer programme (4Ps) benefits even though more than three-quarters of the households of survey participants were living under the poverty line and supposed to be eligible [9]. Finally, a study conducted in Cambodia showed that less than 15% of people with TB involved in the study were enrolled in the Health Equity Fund. Of them, 55% benefitted from free services at governmental health centres; however, no patient in this study received subsidy or reimbursement for their non-medical costs through this scheme [10].

Finally, several implementation issues emerged from the desk review, including: (1) programme sustainability (for example, the Child Money Programme in Mongolia and the Health Equity Fund in Lao PDR); (2) the ascertainment of eligibility—especially for disability grants—in Lao PDR, Cambodia, and Viet Nam; and (3) the need for registration and often payment of fees to access existing benefits, for example, for PhilHealth in the Philippines and the social health insurance in Viet Nam.

As for TB-specific programmes, except for Cambodia, all countries provided some form of TB-specific SP benefits at the time of the assessment. In these countries, these programmes were mainly designed to support people with MDR-TB and, in Mongolia, children with TB as well. Benefits mainly included cash and nutritional support to defray transport, food, and sanatoria visits. In Viet Nam, the National Tuberculosis Programme (NTP) established the PASTB charity fund in 2018 to reduce financial barriers to TB care and support treatment adherence. Since its introduction, PASTB has donated more than half a million USD to people with TB. This programme not only supports the treatment and care of TB patients but also helps mitigate the catastrophic costs that may be incurred, providing holistic support as outlined in the End TB Strategy. The delivery of PASTB is also closely related to Social Health Insurance coverage, as the funds from PASTB are often used to procure health insurance cards that enable registration onto the Social Health Insurance programme [11].

## Existing barriers to access social protection experienced by people affected by TB

Across the five countries, common barriers emerged with varying intensity. Lack of awareness and financial or geographic constraints were reported in all settings; complex administrative procedures and unclear eligibility criteria in four; stigma and social exclusion in four; and programme fragmentation and poor intersectoral coordination in three. These patterns indicate systemic weaknesses in referral pathways between health and social protection sectors and highlight the need for harmonised, one-window enrolment approaches (Table 2).

**Table 2:** Barriers to access to social protection by country

Barrier category	Type of barrier	Mongolia	Lao PDR	Philippines	Cambodia	Viet Nam
Supply-side	Eligibility criteria or assessment of eligibility criteria	●	●	●		●
	Complex and/or inefficient administrative process		●	●	●	●
	Programmes fragmentation	●		●		
	Poor communication/lack of coordinating body	●	●		●	
	Financial constraints/sustainability	●		●		
Demand-side	Social determinants of access*	●	●	●	●	●
	Lack of awareness	●	●	●	●	●
	Stigma	●	●	●		●

^*^Including illiteracy, lack of income to pay contributory schemes, living remotely and not support to cover transport costs

### Supply-side barriers

1. Eligibility criteria or assessment of eligibility criteria.

Some people affected by TB are excluded from the available SP programmes because they do not meet the eligibility criteria, or because there is lack of clarity or transparency in the eligibility assessment.

In Mongolia, for example, the eligibility for the disability grant is often left to the scrutiny of the physician examining the person with TB, who has the ultimate responsibility to raise the case to the welfare and labour commission. Additionally, in order to access the Food Stamp programme, people with TB (like any other applicant) must contact a social worker and confirm their household’s livelihood is affected enough by TB that they fall into the eligibility criteria applied.

In Lao PDR and the Philippines, clinics and clinicians can facilitate access to social health insurance for TB patients by certification of a doctor, but only if they meet the eligibility requirements under the two programmes Health Equity Fund or PhilHealth, and only for the duration of their TB treatment. Further, because people affected by TB are often not working in the formal sector, they are not entitled to receive the social health insurance provided through PhilHealth after completing TB treatment. Conversely, they may not be sufficiently poor to qualify for social security benefits via the Health Equity Fund.

Likewise, in Viet Nam, people with TB who are able to access TB care services might not be poor enough to meet the eligibility of most social assistance programmes available in the country that are means-tested (i.e. not universal but targeted only to people living below a certain poverty or income threshold). Paradoxically, informants from Viet Nam also reported that people with TB who are the least likely to access TB services are often, in fact, those most in need of SP. In other words, since they are invisible to the health services, they are also challenging to refer to the SP services.

[…] *If we don’t do intense active case-finding in the community, many people with TB remain undetected and those missing are usually the poorest of the poor who don’t have the means or the information to go themselves and actively access the TB and SP services. There is a bias here […] (Stakeholder, Viet Nam)*

2. Complex or inefficient administrative process.

The enrolment and benefits disbursement process is often lengthy and complicated by administrative rules that are hard to navigate for disadvantaged groups like TB-affected populations.

Both Cambodia and Lao PDR reported the lack of an identification card or other essential documentation and the complex administrative process as key barriers to accessing existing programmes. In the Philippines, PhilHealth funds are delivered to the facility and not to the individuals; key informants reported challenges from facilities in being adequately and timely reimbursed due to the existing large administrative burden. In Viet Nam, people suffering from TB-associated disabilities often lack the needed documentation (e.g. birth certificate) and must go through long scrutiny to certify the severity of their disability status and that this disability resulted from TB.

3. Programme fragmentation.

There may be multiple and redundant sources of SP benefits, each with different eligibility criteria and administrative processes. This can ultimately increase confusion among beneficiaries and discourage enrolment, especially among households and individuals already struggling because of TB.

Mongolia and the Philippines reported that despite the significant range of schemes available, most of them are fragmented, often overlapping or with conflicting or unclear rules for enrolment and do not benefit a large proportion of the people in need, let alone high TB risk groups such as refugees, stateless people, and people experiencing homelessness, who are also particularly at risk of developing TB.

4. Poor communication/lack of clear guidance.

Both these issues fall under the broader barrier of lack of multisectoral collaboration across the TB and SP sector, making it difficult to establish effective referral systems or understand the reciprocal needs and the potential mutual benefits resulting from a closer collaboration.

Despite the increasing adoption of the principles and platforms underlying the WHO Multisectoral and Accountability Framework for TB (MAF-TB) [12], the existence of poor multisectoral collaborations across the TB and social protection sector was a recurrent theme, and it was mentioned by all countries. Mongolia, Lao PDR, and Cambodia went more specific and argued how—despite the positive will—multisectoral collaboration is often hampered by the frequent changeover of political positions. Stakeholders further identified that the lack of institutional communication channels often results in unclear and not well-implemented guidelines to establish effective referral systems.

5. Financial constraints and limited sustainability.

The provision of SP to people affected by TB is often seen as unfeasible either because of the limited fiscal space to expand the scope and remit of existing schemes or because most of the TB-specific SP programmes rely on external funding.

Stakeholders from both Mongolia and Lao PDR raised concerns about the long-term sustainability of existing programmes, as most of them rely on external donors such as the Global Fund, the Red Cross, and the Asian Development Bank.

### Demand-side barriers

1. Lack of awareness.

People affected by TB may not know the existence of programmes accessible for them, be unaware of their entitlement to receive SP benefits, or struggle to understand the enrolment process.

All countries reported a lack of awareness about existing SP schemes as a critical and most relevant factor currently hampering access to SP from people affected by TB:

“*[…] if we could get the information into those [isolated geographical] areas then I think that would really help in improving the access of people to SP. When they know what they need, and if they know where to go, which office to seek for help, then I think that will be of most value really to their treatment.” (Key informant, Philippines)*

This barrier seems to exist even for flagship and popular programmes like the 4P programme:

*“[…] the household will only learn about it if the social worker *goes* to their house, it is not announced, and it depends on the local chief executive, if they want to promote 4Ps, it depends if they ask the social workers to do this, but if it’s not announced, they won’t know about it.” (Key informant, Philippines)*

Under lack of awareness, most respondents have also mentioned a lack of knowledge about TB and the actual SP needs of people affected by TB among SP providers.

2. Social determinants of access.

While all social assistance programmes are virtually free at the point of use, the process of obtaining benefits may hide economic costs that are unaffordable for recipients, including transportation to reach the service point, income loss, and waiting times. Also, some health insurance schemes are either available only to employed people or may require some enrolment fees that are unaffordable for the poorest segments of the society, which are also those typically affected by TB. These barriers can be worsened when they intersect with other challenges like illiteracy, disability, and living in geographically isolated areas.

All countries involved in this SP assessment reported social determinants as a key barrier to access. In Mongolia, Lao PDR, and Viet Nam, for example, access to existing social health insurance schemes is hampered by their contributory nature (i.e. up to a minimum payment of 99,000 LAK (approximately 5 USD) in Lao PDR and approximately 4.5% of the monthly minimum wage in Viet Nam).

Because of illiteracy and language barriers, several ethnic minority groups living in Lao PDR and the Philippines remain excluded from the health insurance scheme despite the efforts made by the Ministry of Health. Finally, distance from the site of enrolment/registration was also indicated as a key barrier to access to SP, especially from Lao PDR, Cambodia, and the Philippines respondents. Stakeholders from Viet Nam stated that barriers to access to SP are exacerbated for people suffering from TB-associated disabilities.

3. Stigma.

People with TB may go through stigmatising or self-stigmatising experiences because of their TB status and/or because beneficiaries of a form of SP. Discrimination linked to both conditions can be mutually reinforcing and can discourage people affected by TB from claiming the support they are entitled to.

With the exception of Cambodia, stigma was a recurrent theme in all expert consultations: key informants from Mongolia reported that when people affected by TB apply for SP benefits, they are often told to wait until the end of the queue. In Lao PDR and the Philippines, despite the existence of legislation and norms to protect people with TB from any form of discrimination, people are still afraid to disclose their TB and HIV status and to be further stigmatised if applying for any form of SP benefit.

## Solutions to enhance access to social protection for people affected by TB

Key informants proposed a list of possible solutions to address the specific barriers operating in their settings (Table 3). Some of these solutions were reported consistently across all countries and can inform future policies and implementation measures to progress towards universal SP coverage among people affected by TB in the Western Pacific region (Box 2):

1. Expand access to SP by advocating for the inclusion of all people affected by TB into existing National SP Programmes and/or by extending the eligibility of TB-specific programmes to people with drug-susceptible TB.

**Table 3:** Country-specific suggestions to enhance access to social protection from people affected by TB

Suggestion	Mongolia	Lao PDR	Philippines	Cambodia	Viet Nam
Extend and improve TB-specific benefits to all people with TB	●	●	●	●	●
Advocate for better inclusion of people affected by TB into existing schemes	●	●	●	●	●
Intensify awareness campaign/development of information material	●	●	●	●	●
Enhance training among social protection and TB care staff			●		●
One-window approach/harmonisation of programmes	●	●	●	●	●
Streamline administrative requirements					●
Assess routinely social protection needs and eligibility of people affected by TB	●				●
Reduce stigmatisation			●		●
Link TB survivors to group savings initiatives					
Strengthening multisectoral collaboration	●	●		●

**Box 2:** Policy and implementation implications to achieve universal social protection coverage for people with TB in the Western Pacific Region

1. Extend TB-specific benefits beyond multidrug-resistant TB to include all people with TB to support them through the various stages of the pathway to TB care, while leveraging TB-sensitive schemes to address the social determinants of TB and offset indirect expenses such as food, transport, and income loss.	
2. Invest in the development of culturally appropriate, non-stigmatising awareness campaigns and user-friendly material (including digital tools) targeting people with TB and their affected families. At the same time, ensure that both healthcare and SP personnel receive adequate training on how to identify and respond to the SP needs of people affected by TB.	
3. Institutionalise bilateral TB–social protection referral systems with clear operating procedures, shared registers, and designated focal points; adapt Mongolia’s maternal and community touchpoints and Viet Nam’s social-worker model as practical examples.	
4. Reduce fragmentation and administrative burden through a one-window enrolment and timely reimbursement mechanism, and plan for sustainable domestic financing to ensure continuity and sustainability of benefits.	

One key informant from Cambodia suggested that the NTP should work more closely with the local authorities and NGOs (such as KHANA) to facilitate the registration and enrolment into the ID Poor system, which is needed to be eligible for the Health Equity Fund. According to the Decision No. 15/QD-QHTNBCTBL dated April 20, 2020 of the Board of Directors of the Fund to Support Patients to Conquer Tuberculosis, Viet Nam is also committed to buying health insurance cards for all cases of TB who do not have health insurance cards and have registration documents according to the Fund’s regulations. In the Philippines, PhilHealth is also presently developing a plan to expand TB outpatient services for people with drug-sensitive TB.

Key informants from Cambodia noted that their National Tuberculosis Strategic Plan already recommends that all people with TB and their affected families receive a compensation of all the indirect costs sustained during TB care and all health care providers involved are reimbursed for providing TB care. Viet Nam went further by proposing to survey people affected by TB-related disabilities to understand their actual eligibility for disability grants.

2. Intensify training and awareness campaigns.

All countries concurred on the need to raise knowledge and awareness among people affected by TB about available SP schemes and that healthcare as well as SP providers should be trained to become aware of the health and SP needs of people with TB. Countries could develop culturally appropriate, non-stigmatising educational material or user-friendly digital platforms (i.e. via SMS, social media) provided to people with TB and their households at the entry point of care to enable them to access information on what TB-sensitive and TB-specific schemes are available for them, where to register for suitable SP schemes, and where to gather additional information about the enrolment process. Likewise, SP key staff and personnel can receive education and training on TB and the needs and challenges experienced by people with TB.

3. Enhancing harmonisation and referral system across sectors.

The harmonisation of programmes appeared vital to establish effective and simple bilateral screening procedures across the TB and SP sector. Possible entry points for establishing effective bilateral referral systems are available in Mongolia, as illustrated in Box 3. Since 2018, Viet Nam has supported people with TB/MDR-TB in accessing the available SP services through its network of social workers who are purposely trained to obtain information about services and policies and to introduce clients to available policies, services, and resources from individuals and organisations so they can access these resources. Box 4 illustrates how this network of social workers operates to support people with TB/MDR-TB (unpublished data). The same could be achieved in Cambodia through initiatives like the Community Health Workers Village Health Support group, the TB-DOT watchers, together with several NGOs (namely one called KHANA). These figures could be effectively trained to identify social protection needs among people with TB and screen their eligibility for suitable SP programmes, support them at navigating administrative processes and thus facilitate their enrolment into existing schemes.

**Box 3:** Entry points for an effective TB-social protection referral system in Mongolia

In Mongolia a suitable entry point for this approach are pregnant women who are all offered a symptom-based TB screening and are all eligible for the Allowance for Mothers and Children]. Another entry point for establishing a referral system is the community-based DOT programme provided by the Mongolian Anti-Tuberculosis Association.	
Lay health workers delivering anti-TB drugs and also providing food support could screen people with TB for their eligibility to SP schemes available in the country. Finally, primary care providers to people with TB with children and/or to children with TB may simply confirm who is already receiving benefits from the Child Money Programme and support the access for those who are not yet enrolled.	
This referral effort seems feasible and likely to involve only a small number of households affected by TB, since according to UNICEF, this programme already covers 97% of households in the country.

**Box 4:** Social protection support to TB/MDR-TB through a network of social workers in Viet Nam

From 2018 up to the time of this assessment, MOLISA has piloted the provision of social support services for people with TB/MDR-TB in the community in 5 provinces/cities including: Hoa Binh, Khanh Hoa, Da Nang, Thanh Hoa, and Vinh Long.	
Services provided include:	
(1) Consulting patients to comply with treatment and avoid treatment withdrawal; periodic monitoring, contact, and information exchange;	
(2) Nutritional care, support for purchasing health insurance cards, and support for livelihood models for patients from poor and near-poor households among managed patients;	
(3) Screening, identifying, and classifying groups vulnerable to TB and at risk of contracting TB at social assistance facilities, and carrying out communication work to raise awareness of TB prevention.	
In 2021, in 5 provinces/cities piloting the social support model for TB/drug-resistant TB patients, overall 2,000 people were consulted and examined for suspect TB. Of these, 570 received case-based counselling and social support such as nutritional care, 67 people received support to purchase the health insurance card and emplopyment support was provided to 36 people.	

Finally, in the Philippines, the 4Ps programme includes an educational module on TB symptoms as well as the pathway to TB care. If households enrolled in the programme have a person with presumed TB, social workers refer them to the local TB services. However, there is currently no data on the number of people with presumed TB who have started the pathway to TB care as a result of this referral mechanism.

## Lessons learned and way forward

Overall, this SP baseline assessment reveals a diverse array of TB-specific and TB-sensitive programmes across the countries studied. However, neither the TB-sensitive nor the TB-specific programmes seem sufficient to meet the needs of people affected by TB. Moreover, they all appear poorly accessible to people with TB. A broad range of demand- and supply-side barriers seems to explain the current picture. In response to this social protection landscape, countries have formulated three priority solutions, including:

- Enhancing the inclusion of TB-affected people into existing TB-sensitivity schemes while expanding the benefits of TB-specific programmes to *all* people affected by TB;
- Raising knowledge and awareness about SP among people affected by TB and TB care providers; and
- Improving programme harmonisation and referral systems across the TB and SP sector.

These solutions align well with the strategic actions outlined in a recent commentary from Fuady et al. [13]. Thye also reflect consistently the vision and principles underlying the Regional Framework for Reaching the Unreached in the Western Pacific Region [14]: firstly, the road map for better inclusion of SP in the programmatic response to TB is not beyond the scope and remit of NTPs and Ministries of Health: they can, in fact, advocate for better TB sensitivity of existing programmes, work towards building or further consolidating existing dialogue with the SP sector, facilitate the development of shared understandings of the different terminologies, and agree on common strategic goals and objectives, co-develop information material on programmes’ availability and enrolment procedures [6]. NTPs can also develop tools to accurately identify those in need of SP benefits [15] and design and deliver ex novo TB-specific SP programmes.

Secondly, the implementation of concrete multisectoral collaborations across the health and SP sector is the backbone of all the proposed solutions; however, efforts should be made to understand what collaborative models are more likely to be effective and sustainable, and what additional human and financial resources are needed to create concrete synergies across sectors.

Thirdly, no investment in SP programmes is likely to have an impact on TB, or other health outcomes, if no sustained efforts towards universal health coverage and health system strengthening are made, especially in geographically isolated areas. Progress towards more inclusive and responsive SP programmes needs to happen along with the improvement of the pathway to TB care, including the scale-up of new technologies and services in both public and private sectors, the decentralisation of health care services, the enhancement of active case-finding and treatment adherence enhancement strategies such as counselling, peer support, or mental health care support.

This SP baseline assessment proved to be feasible and acceptable from the five countries involved in this initiative; however, efforts should be granted to convert this approach into a generic protocol that countries (within and outside the Western Pacific Region) can adopt periodically to obtain more accurate data on the availability of SP programmes and their health sensitivity, to assess SP needs, the context- and disease-specific barriers, and facilitators that affect access and impact of those programmes. To ensure better sustainability and efficiency, SP baseline assessments could be mainstreamed as part of regular TB programme reviews.

## Conclusions

This SP baseline assessment provided countries with a valuable basis for the future planning and implementation of SP programmes for people affected by TB. While a more systematic and periodic assessment is needed to appraise the accessibility and responsiveness of existing SP programmes, the lessons learned in this study have the potential to inform actions to accelerate progress to end TB. Furthermore, these insights are not only relevant to TB but also have broader applicability, offering a potential roadmap suitable for various diseases of poverty. Overall, these findings advocate for necessary policy reforms and programmatic adjustments to ensure SP programmes become an integral part of the public health response to TB and other global health challenges in the region, thus contributing to the health equity and social justice vision and principles underlying the broader Regional Framework for Reaching the Unreached [14].

## Supplementary Information

**Additional file 1.**

**Additional file 2.**

**Additional file 3.**

## Data Availability

All data generated or analysed during this study are included in this published article [and its supplementary information files].
