# Supplementary material for: Towards universal social protection for people affected by tuberculosis in the Western Pacific Region: a social protection baseline assessment and policy entry points
Source: Trop Med Health. 2026 Mar 12;54:47. doi: 10.1186/s41182-025-00887-2 (PMC12980936; doi:10.1186/s41182-025-00887-2)
Supplement: Supplementary file 1 — Additional file 1. [file 41182_2025_887_MOESM1_ESM.docx]

**Appendix 1 - The social protection baseline assessment: summary of the methodological approach used**

The approach taken for the social protection baseline assessment in Mongolia, Lao PDR, the Philippines, Cambodia, and Vietnam largely draw from content of the WHO-ILO Guidance on Social Protection for People affected by TB (https://www.who.int/publications/i/item/9789240089327). This approach encompassed three steps, including 1. The establishment of a coordination team to lead the work; 2. A desk review of the existing social protection landscape (including TB-sensitive and TB-specific programmes, Box 1) and 3. A multisectoral expert consultation with the relevant stakeholders from the TB and social protections sector, including TB care providers, National TB programmes managers and staff, TB survivors and social protection implementers from governmental and non-governmental institutions. These experts were identified by the National TB Programme in each country in consultation with WHO Headquarters. Consultations were held only when at least one representative for each stakeholder group above was able to ensure his/her participation.

The desk review was not systematic nor a scoping review, hence the scope of work was not to cover all the existing available evidence; rather, the desk review aimed to gathered information from the most relevant resources including published and unpublished social protection reports, as well as peer-reviewed scientific literature and purposively appraise them using a list of a priori selected criteria. Specifically, references were deemed of interest only when the source could be verified and priority was given to governmental reports, evidence gathered from the International Labour Organization (ILO) and documentation recommended by experts identified among the stakeholder group. Programmes deemed of relevance for people affected by TB were appraised in terms of the following criteria: i. coverage, ii. adequacy (*i.e.* the extent to which the size and type of benefits provided were sufficient to meet the needs of people affected by TB and their household members) and iii. implementation and operational aspects.

The multisectoral expert consultation aimed to complement the desk review by investigating further the TB inclusiveness and responsiveness of the priority programmes identified. Key stakeholders identified by the coordination team were invited to take part to an online consultation to:

1. Comment on the key findings of the desk review;
2. Discuss the actual or perceived barriers to access experienced by people affected by TB in the country. Barriers to access were divided into supply-side and demand-side barriers; and
3. Brainstorm possible solutions to overcome the identified barriers, enhance access, and ultimately maximise the impact of social protection programmes on people affected by TB.

Participants in the consultation were invited to join the discussion from remote via Zoom. Prior to the consultation, key informants received a list of themes and relevant knowledge gaps to be discussed that emerged from the synthesis of findings of the desk review. To facilitate the discussion, each consultation was structured around three main themes: a. Key TB-affected populations (high-risk groups in their setting); b. Existing social protection programs available or potentially available to TB patients and barriers to access. C. programmatic solutions to enhance access to social protection. For each theme, a list of questions was derived as informed by the findings emerged by the desk review in each country. An example of key guiding questions asked to participants is listed below:

- Is there sufficient capacity amongst current TB programming to integrate social protection programs, including at the human resource level?
- Are there key loci for delivery of integrated programs through a single window approach?
- Do you consider the existing TB-specific programmes as effective in meeting the SP needs of people affected by TB in your country?
- Is there any form of established collaboration between the NTP and possible partners for social protection development in your country?
- Is the National TB programme known to key actors from the SP sector?
- Is there generally felt to be sufficient evidence to prioritise the implementation of social protection for people affected by TB in your country?
- Have the key SP needs of people affected by TB already been identified?
- Which specific populations are viewed as most at risk of TB and are they likely to meet the eligibility criteria for existing social protection programmes?
- In your view as TB survivor/TB civil society representative, what are the key barriers do people with TB face in accessing social protection?

Each consultation lasted for approximately 90 minutes and a translator was present at each session. With the approval of stakeholders, the content of each consultation was recorded and transcribed. Emerging themes were synthetised and translated into over-arching/cross-country and country-specific suggestions to inform the future planning and implementation of social protection programmes for people affected by TB in these settings.
