# Supplementary material for: Towards universal social protection for people affected by tuberculosis in the Western Pacific Region: a social protection baseline assessment and policy entry points
Source: Trop Med Health. 2026 Mar 12;54:47. doi: 10.1186/s41182-025-00887-2 (PMC12980936; doi:10.1186/s41182-025-00887-2)
Supplement: Supplementary file 2 — Additional file 2. [file 41182_2025_887_MOESM2_ESM.docx]

**Appendix 2 – List of available TB-sensitive and TB-specific social protection programmes available in the five countries at the time of the social protection baseline assessment.**

Overall, all five countries in the social protection baseline assessment had several available social protection programmes, including potentially TB-sensitive (Table 1) and TB-specific social protection schemes (Table 2).

Under this group, the desk review identified the existence of schemes aiming at managing economic and social shocks as well as reducing the vulnerability of critical stages across the life course, including childhood, motherhood, unemployment, sickness, disability and old age (Table 1). All countries offered some form of cash transfer – conditional or unconditional – as well as food support or school feeding programmes. Additionally, all countries but Mongolia had a programme aimed at covering health insurance fees for poor households and all countries, but Lao PDR offered some kind of voucher or subsidy programme. The Philippines, Cambodia, and Viet Nam additionally offer conditional cash transfer and employment programmes, and only Cambodia had a programme covering employment insurance for disadvantaged people.

All countries except for Cambodia also offered TB-specific social protection for multidrug-resistant (MDR) TB, and Mongolia additionally offered these for children with TB (Table 2).

**Table 1 – Main TB-sensitive social protection programmes available in the five countries at the time of the assessment.**

|  | Unconditional cash transfer | Conditional cash transfer | Subsidies & Vouchers | Food Support &  School Feeding Programmes | Social Health Insurance | Social Employment Insurance | Employment Programmes |
| --- | --- | --- | --- | --- | --- | --- | --- |
| Mongolia | Child Money Programme; Allowance for Mothers & Children incl Mother Heroes;  Social Welfare Pensions & Allowances for the Elderly and Disabled; Carer's Allowances (various) | -- | Food Stamps;  Transport & Food Subsidies (people with disabilities) | School Feeding Programme | -- | -- | -- |
| Lao PDR | Disability Grant | -- | -- | National School Meals | Health Equity Fund | -- | -- |
| Philippines | Social Pension for Senior Citizens | 4Ps (Pantawid Pamilya, Pantawid  Pamilyang Pilipino Programme) | Rice Subsidy Programme | School Feeding Programme | National Health Insurance  (enrolment in PhilHealth) | -- | Integrated Livelihood &  Employment Programme (DILEEP) |
| Cambodia | Disability Allowance;  Cash Transfer Programme  (pregnant mothers & children under 5);  Social welfare programme for vulnerable children | School Attendance Scholarships | Reduced electricity tariff | School Feeding Programme | Health Equity Fund | National Social Security Fund | Productive Assets & Livelihood Support (PALS); Second Chance  (vocational education: TVET) |
| Viet Nam | HIV/AIDS Allowance; Child benefits;  Disability benefits;  Social Pension Scheme;  Cơ hội thoát nghèo truyền kiếp  (Opportunity to move out of  intergenerational poverty) | School Stipends | Electricity & Fuel Subsidies | Food support for border households | Social Health Insurance | -- | Livelihood support programme for farmers and smallholders |

**Table 2 – TB-specific social protection programmes available in the five countries**

|  | **Cash support** | **Subsidies & Vouchers** | **Food support** | **Target population** |
| --- | --- | --- | --- | --- |
| **Mongolia** | -- | Bus card;  Sanatoria visits; Summer camps for children | Nutritional support | MDR-TB, children with TB |
| **Lao PDR** | Cash support for food and transport | -- | -- | MDR-TB |
| **Philippines** | Cash support for food and transport | -- | -- | MDR-TB |
| **Cambodia** | -- | -- | -- |  |
| **Viet Nam** | Patient Support to Fight TB (PASTB) | -- | -- | MDR-TB |
