# Supplementary material for: Towards universal social protection for people affected by tuberculosis in the Western Pacific Region: a social protection baseline assessment and policy entry points
Source: Trop Med Health. 2026 Mar 12;54:47. doi: 10.1186/s41182-025-00887-2 (PMC12980936; doi:10.1186/s41182-025-00887-2)
Supplement: Supplementary file 3 — Additional file 3. [file 41182_2025_887_MOESM3_ESM.docx]

**Appendix 3 – Key features of the identified TB-sensitive programmes by country at the time of the assessment**

| **Country** | **Programme** | **Brief description** | **Coverage** | **Adequacy** | **Implementation issues** |
| --- | --- | --- | --- | --- | --- |
| Mongolia | Child Money programme | It provides universal benefit for households with children. By far the largest social protection programme active in the country and the largest share of income from social protection received by poor individuals in Mongolia. | It is estimated that 98% of the households in Mongolia are covered.  No data on the proportion of households affected by TB covered by the programme. | During the COVID-19 pandemic, the value of the benefit delivered by the Child Money Programme was quintupled to 100,000 Mongolian Tugrik (approximately $28 US dollars).  This top-up has been cited as one of the most effective social protection responses to the pandemic, and simulation scenarios suggest the amount of money provided was adequate to meet the additional needs for care. | It requires little administration and infrastructure (mainly because its nearly universality nature that does not require any targeting effort).  The programme is delivered by direct bank transfer and any eligible household can apply at any bank.  Sustainability issues linked to the external source of funding. |
|  | Disability social pension | Universal access to people incurring any form of disability, either temporary or permanent, through a social pension. | It is considered the primary route of support for people affected by TB.  2500 individuals with TB were included in the programme at the time of assessment under the designation of people living with disabilities. | Recipients receive a pension of approximately 288,000 Mongolian Tugrik per month as of January 2023 (approximately $84 US dollars).  Amount of money and coverage was considered insufficient at the time of assessment to cover the full needs of people with TB. | For people working in the formal sector a certain number of years of work is required. Further, extent of disability must be ascertained. |
| Lao PDR | Health Equity Fund (HEF) | It provides health insurance to poor households in the country.  HEF is targeted to poor households in certain districts of Lao PDR using a series of eligibility requirements based on and income threshold and the lack of key assets such as clothes and rice and others. | There has been notable expansion of the National Health Insurance (NHI) coverage in recent years, increasing from 11 per cent in 2008 to 94.5 per cent in 2023 according to the Ministry of Health^[[1]](#footnote-1)^. This was achieved by adopting a mix of funding mechanisms, combining social contributions with tax-based ﬁnancing and direct co-payments.  No data on the proportion of households affected by TB covered by the programmes. | The programme aims to provide coverage at the primary care level to individuals not already covered by the NHI, as well as covering transport and food costs.  While coverage has increased, household spending on health care has not necessarily improved, with a doubling of the proportion of households suffering catastrophic health spendings (3.0 per cent in 2007 to 6.7 per cent in 2019)^^[[2]](#footnote-2)^^.  In this country people with TB begin their diagnosis pathway at district level facilities but must be treated at tertiary level which may rapidly increase costs for transportation and access to the services required that people with TB. are often unable to afford.  Several TB services related to treatment or more complex TB are provided at tertiary levels and are not covered by the health insurance  Finally, many services related to treatment of more complex TB are not covered by the health insurance. | The programme is managed by the Ministry of Health and a variety of international actors such as the Red Cross and the Asian Development Bank fund the scheme^^[[3]](#footnote-3)^^.  It has been highlighted that the financing of this scheme from grant aid is likely to be unsustainable in the long run. There has been a recent co-financed investment into further improving access to health and nutrition from the Global Fund and the World Bank but the impact of this investment has yet to be assessed^^[[4]](#footnote-4)^^.  The financial sustainability of the HES is also fragilized by irregular, insufficient and unpredictable government resources allocations and increasing costs of care.  The current health insurance programme requires a flat contribution for both inpatient and outpatient care dependent on location (clinic vs hospital, e.g.)^^[[5]](#footnote-5)^^. |
|  | Disability grant | Lao PDR’s social insurance includes two main disability-related cash supports for insured workers: a disability pension (if the disability is severe) and a lump-sum disability grant (for milder disability)^[[6]](#footnote-6)^. | Although people with TB are eligible to receive disability grant or sickness benefit for 6 months in Lao PDR only 0.4% reported that they received any types of social welfare. | Beneficiaries receive a lump sum of 10 times the reference earnings multiplied by the assessed loss of work capacity. | Eligible people must have an assessed loss of work capacity of less than 41% and at least 12 months of contributions in the last 24 months.  The National Social Security Fund's Medical Committee assesses the loss of work capacity. |
| Philippines | [Pantawid Pamilya Pilipino Program](https://pantawid.dswd.gov.ph/images/stories/pantawidfaq.pdf) or 4Ps | It is the flagship national conditional cash transfer programme of the Philippines, accounting for approximately 0.5% GDP of the country and distributed on the basis of a proxy means test  Benefits are payable to households identified as poor under the NHTS-PR (i.e. the National Household Targeting System for Poverty Reduction), also known as Listahan based on the assessment of the living conditions of potentially eligible households.  Poor households must include either children ages 0–18, or a pregnant woman. | The program benefits about 20% of the population, the majority of the nation’s poor.  A significant proportion of the poor among informal and agricultural sector workers are covered by 4Ps, but precise data about coverage are not available. It is known though that in 2012 73% of 4Ps beneficiaries were agricultural households.  Evidence from the patient-cost survey conducted in this country highlighted that only 1.3% of TB-affected households were receiving 4P benefits even though more than three-quarters of the households of survey participants were living under the poverty line and supposed to be eligible for 4Ps^[[7]](#footnote-7)^. | Benefits consist of a conditional cash transfer of between PHP 500 and PHP 1,400 (US$11–31) per household per month depending on the number of eligible children; and an educational payment of PHP 300 (US$6.60) per child per month for 10 months of the year, up to a limit of three children per household.  The programme also provides free health insurance (as PhilHealth, discussed below), a rice subsidy, microfinance opportunities, and employment-related activities to families with children.  Despite this, actual benefits reportedly received by 4Ps households are significantly lower than the targeted level, at about 11 percent of consumption. | It is administered by the Department of Social Welfare and Development (DSWD) in collaboration with other departments and local government units, and it is supported by the Asian Development Bank, UNICEF, and the World Bank.  Payments are delivered every two months via debit card or mobile payment.  Despite the impressive growth and success of the program, at the time pf assessment the sustainability of strong and positive impacts of 4Ps could not be ensured unless core program functions are improved, including (1) the targeting, (2) the benefit level and conditionalities, (3) the program duration, and (4) payment modality. |
|  | Phil Health | The National Health Insurance Programme of the Philippines is a form of non-contributory health insurance that covers a wide variety of populations within the Philippines.  The programme is targeted through a proxy means test to poor households. | PhilHealth increased its effective population coverage from 73 per cent in 2007 (64.6 million members, including dependents) to 85 per cent of the total population in 2020 (93.3 million beneficiaries).  PhilHealth has successfully extended coverage to more than 18 million workers in the informal economy and their dependents through adapted financing and administrative mechanisms. This significant expansion has led to high health protection coverage in the Philippines.  No data on the proportion of households affected by TB covered by the programmes. | Despite broad population coverage, the burden of OOP health expenditures remains high for Filipino households, underlying the still limited adequacy of the benefits provided.  The programme gives individuals (and in a more limited way, their dependents) access to PhilHealth-accredited health facilities and includes TB treatment and care.  Payments for this programme are delivered to the health facility so that treatment is free at point of care for individuals covered by this health insurance programme.  PhilHealth introduced the TB DOTS outpatient benefit package to deal with the burden of TB. Accredited TB-DOTS centres (public and private) were strategically conceptualized by the Philippine Coalition Against Tuberculosis and PhilHealth to help finance detection and treatment of TB cases by PhilHealth. Only accredited facilities providing TBDOTS treatment are eligible to receive reimbursement from PhilHealth.  By 2020, 20% of all PhilHealth accredited facilities provided the TB-DOTS package.  Theoretically, a payment to the facility is made on each diagnosis of TB, for example. However, data from 2016 suggest that reimbursements were claimed from PhilHealth are claimed only for about 11% of the notified TB patients in 2016^[[8]](#footnote-8)^ | PhilHealth membership registration is required to access benefits, following which each member is provided with a Member Data Record (MDR) and a PhilHealth ID Card, which is also recognized as a means of identification in the Philippines.  A number of barriers to access (see paper 2) results into relatively low utilization rates of Phil Health in the Philippines, particularly among the poor^[[9]](#footnote-9)^. |
| Cambodia | Heath Equity Fund (HEF) | The Cambodia’s HEF is a form of social health insurance that provides access to healthcare for the poor and vulnerable population via non-contributory health insurance.  Beneficiaries, including people with TB, can use health services at public referral hospitals and health centres, as well as additional benefits such as transportation reimbursements and daily food allowances for caretakers | As of 2021 the programme covered approximately 2.9 million people (about 20% of the population).  Only 26% of beneficiaries tend to seek healthcare in the public sector; the remaining proportion relies on the private sector which could amount to elevated costs.  Data from one study suggest that only 29 people with TB (out of the 208 included in the study) were enrolled in the Health Equity Fund. Of them, 16 (55%) benefitted from free services at governmental health centres; however, no patient in this study received subsidy or reimbursement for their non-medical costs through this scheme^[[10]](#footnote-10)^. | The current level of protection from the HEF scheme is a good start but by itself would not be sufficient for Cambodia to achieve UHC.  Sick individuals with HEF benefits still have to pay some level of out-of-pocket expenditures for hospitalization in public hospitals suggesting that benefits provided are not adequate to defray all health costs.  Given the magnitude of TB-associated catastrophic costs (medical costs for TB care exceeding 10% of the annual household income), this coverage might not meet the needs of many TB patients. | A key tool for enrolling in the HEF is the Identification of Poor Households Programme (IDPoor), a nationwide poverty identification system that has allowed for better targeting of vulnerable households. It relies on a combination of proxy means testing and community-based targeting, to ensure adequate identification. Households identified are given an IDPoor card which gives them access to SP programmes across multiple sectors.  As the IDPoor system has been mostly spread in rural areas[]](https://paperpile.com/c/bFrPp1/EeaE), individuals facing increased TB transmission in urban areas might not be reaping the benefits of the programme if they develop TB disease. |
|  | Disability allowance | Since 2013, this programme has provided unconditional cash transfers to people living with disabilities who are poor, elderly or have no supporting families, verified by local authorities. | Coverage is nominally nationwide.  No data on the proportion of households affected by TB covered by the programmes. | Beneficiaries receive a monthly benefit of $5 US dollars. | According to the National Social Protection Policy Framework 2016-2025, the scheme focuses on people with disabilities who are members of households holding an ‘ID poor’ card.  TB does not generally fall under the criteria for access to this allowance unless severe disease or complications have been incurred. |
| Viet Nam | Social Health Insurance | The social health insurance programme of Viet Nam is non-contributory.  The non-contributory component of the SHI applies to many different social groups including poor people, ethnic groups, children, and other social assistance beneficiaries  SHI provides access to healthcare services at varying subsidy rates, primarily covering poor individuals, minorities, elderly above the age of 80 and children under 6 by 100%, including ambulatory care, rehabilitation, and advanced diagnostics. | In 2022, the programme covered 92% of the population which the government and appears on track to reach the goal of covering 95% of the population by 2025 moving towards universal healthcare.  No data on the proportion of households affected by TB covered by the programmes. | This programme might provide support against the catastrophic costs faced by TB patients by reducing out-of-pocket expenditure, as it is meant to directly cover TB related costs. However, Enrolment in the SHI after a TB diagnosis does not guarantee immediate access to the benefits, with 20% of the treatment duration remaining uncovered when the highest costs are incurred. | Access is granted after registration with the Provincial Social Security where a card is issued; for near-poor people, they must first pay their premium contribution (4.5% of monthly minimum wage) before receiving their cards. |
|  | HIV/AIDS allowance | This is an unconditional cash transfer programme providing social assistance to people living with HIV/AIDS who are unable to work and are considered poor since 2004.  This program is potentially highly TB-sensitive as it targets a population at high risk of developing TB | Available data indicate that as of 2014 approximately 4,000 people were enrolled in this programme.  No data on the proportion of households affected by TB covered by the programmes. | Benefits amount to VND 675,000 (approximately $25 US dollars) per month for children under 4 years of age and VND 540,000 per month (approximately $20 US dollar) for children over 4 years of age; VND 405,000 per month (approximately $15 US dollar) for people over 16 years of age. | Payments are made by local officials. |
|  | Disability benefits | It is an unconditional cash transfer programme addressing people living with disabilities and pregnant women. | As for 2014, the number of beneficiaries were as follows:  Disability benefits: 745,265 recipients  Serious mental illness benefits: 174,254 recipients  Households with two or more people living with disabilities: 22,939 recipients.  No data about coverage among people affected by TB-related disabilities | The program provides a variable amount of benefits (up to VND 675,000, approximately $25 US dollars) based on the severity of the disability or the number of children being raised. | A medical board from the Ministry of Health assesses the degree of disability of potential recipients. |
|  | “Cơ hội thoát nghèo truyền kiếp”—“Opportunity to Move out of Inter-generational Poverty” | Since 2015, the "Opportunity Program" has been aiming to reduce and improve human development outcomes for children by providing cash transfers to the head of household,  There are a few soft conditionalities pertaining to child health and education, including immunisation, growth monitoring, nutrition counselling, school enrolment and attendance.  As individuals belonging to ethnic minorities and experiencing poorer socio-economic conditions can be more vulnerable to TB disease, this programme can be considered potentially TB-sensitive | As of 2017 there were approximately 400,000 beneficiaries within four provinces (Hà Giang, Quảng Nam, Trà Vinh and Lâm Đồng), as these contain a large share of households that are from ethnic minority backgrounds and to ensure a diverse mix of geographic locations.  No data on the proportion of households affected by TB covered by the programmes. | The monthly benefit varies according to the number of beneficiaries per household and also according to the schedule of payments for each type of benefit.  The average annual benefit amount is VND 1,360,000 (approximately $52 US dollars). | The benefit payment is operated by a service provider (VNPOST).  The programme has a maximum duration of nine months for pregnant women, nine months per year for out-of-school children aged 3-16 years, and 12 months per year for children under 3 years old. |

1. https://iloglobalprogramme.social-protection.org/gimi/ShowCountryProfile.action?iso=LA [↑](#footnote-ref-1)
2. https://www.who.int/publications/i/item/9789290620037 [↑](#footnote-ref-2)
3. https://www.who.int/laos/news/detail/08-11-2021-who-continues-to-support-the-ministry-of-health-to-improve-equitable-access-to-healthcare-services-through-strengthening-social-health-protection-schemes-in-lao-people-s-democratic-republic [↑](#footnote-ref-3)
4. https://www.theglobalfund.org/en/updates/2024/2024-04-17-asian-development-bank-global-fund-join-forces/ [↑](#footnote-ref-4)
5. Bodhisane S, Pongpanich S. The impact of National Health Insurance upon accessibility of health services and financial protection from catastrophic health expenditure: a case study of Savannakhet province, the Lao People's Democratic Republic. Health Res Policy Syst. 2019 Dec 16;17(1):99. doi: 10.1186/s12961-019-0493-3. PMID: 31842882; PMCID: PMC6915990. [↑](#footnote-ref-5)
6. Chittamany P, Yamanaka T, Suthepmany S, Sorsavanh T, Siphanthong P, Sebert J, et al. First national tuberculosis patient cost survey in Lao People's Democratic Republic: Assessment of the financial burden faced by TB-affected households and the comparisons by drug-resistance and HIV status. PLoS One. 2020;15(11):e0241862. [↑](#footnote-ref-6)
7. Florentino JL, Arao RML, Garfin AMC, Gaviola DMG, Tan CR, Yadav RP, et al. Expansion of social protection is necessary towards zero catastrophic costs due to TB: The first national TB patient cost survey in the Philippines. PLoS One. 2022;17(2):e0264689. [↑](#footnote-ref-7)
8. Wells WA, Stallworthy G, Balsara Z. How tuberculosis programs can navigate the world of social health insurance. Int J Tuberc Lung Dis. 2019 Jan 1;23(1):26-37. doi: 10.5588/ijtld.18.0289. Epub 2018 Nov 13. PMID: 30424825. [↑](#footnote-ref-8)
9. Dayrit MM, Lagrada LP, Picazo OF, Pons MC, Villaverde MC. The Philippines Health System Review. Vol. 8 No. 2. New Delhi: World Health Organization, Regional Office for SouthEast Asia; 2018. [↑](#footnote-ref-9)
10. Morishita F, Yadav RP, Eang MT, Saint S, Nishikiori N. Mitigating Financial Burden of Tuberculosis through Active Case Finding Targeting Household and Neighbourhood Contacts in Cambodia. PLoS One. 2016 Sep 9;11(9):e0162796. doi: 10.1371/journal.pone.0162796. PMID: 27611908; PMCID: PMC5017748. [↑](#footnote-ref-10)
